# Redundancy of IL-1 Isoform Signaling and Its Implications for Arterial Remodeling

**DOI:** 10.1371/journal.pone.0152474

**Published:** 2016-03-31

**Authors:** Marina Beltrami-Moreira, Amélie Vromman, Galina K. Sukhova, Eduardo J. Folco, Peter Libby

**Affiliations:** Division of Cardiovascular Medicine, Department of Medicine, Brigham and Women’s Hospital, Harvard Medical School, Boston, Massachusetts, United States of America; Duke University, UNITED STATES

## Abstract

**Aims:**

Mice deficient in IL-1 receptor 1 (hence unresponsive to both IL-1 isoforms α and β) have impaired expansive arterial remodeling due to diminished expression of matrix-degrading enzymes, especially MMP-3. Emergence of IL-1 as a target in cardiovascular disease prompted the investigation of the redundancy of IL-1α and IL-1β in the induction of MMP-3 and other matrix-remodeling enzymes in human cells.

**Methods and Results:**

Human primary vascular smooth muscle cells (VSMCs) and carotid endarterectomy specimens were stimulated with equimolar concentrations of IL-1α or IL-1β and analyzed protease expression by immunoblot and ELISA. Either IL-1α or IL-1β increased the expression of pro-MMP-3 in VSMCs, facilitated VSMC migration through Matrigel, and induced MMP-3 production in specimens from atheromatous plaques. VSMCs also secreted MMP-1 and Cathepsin S (CatS) upon stimulation with IL-1α or IL-1β. IL-1 isoforms similarly increased MMP-1 and MMP-9 expression in carotid endarterectomy specimens. We examined the expression of MMP-3 and IL-1 isoforms by immunostaining of carotid atheromata, calculated the % positive areas, and tested associations by linear regression. MMP-3 colocalized with IL-1 isoforms in atheromata. MMP-3+ area in plaques positively associated with IL-1α+ (R2 = 0.61, P<0.001) and with IL-1β + areas (R2 = 0.68, P<0.001). MMP-3+ area within atheroma also associated with CD68+ area, but not with α-smooth muscle actin area.

**Conclusions:**

Either IL-1α or IL-1β can induce the expression of enzymes implicated in remodeling of the arterial extracellular matrix, and facilitate human VSMC migration *in vitro*. Human atheromata contain both IL-1 isoforms in association with immunoreactive MMP-3. This redundancy of IL-1 isoforms suggests that selective blocking of one IL-1 isoform should not impair expansive arterial remodeling, a finding with important clinical implications for therapeutic targeting of IL-1 in atherosclerosis.

## Introduction

Many acute thrombotic complications of atherosclerosis result from the fracture of the plaque’s fibrous cap.[1] Proteolytic activity within the atheroma favors degradation of extracellular matrix macromolecules that lend tensile strength to the plaque’s fibrous cap.[2, 3] Interleukin-1 (IL-1), a cytokine implicated in vascular inflammation,[4, 5] induces the expression by cells found in atheromata of several matrix-degrading proteases implicated in lesion remodeling.[6–8]

Although excessive cytokine-induced proteolytic activity in the atheroma associates with risk of plaque rupture,[1] recent work has unveiled an unexpected effect of IL-1 receptor 1 (IL-1R1) on the preservation of luminal caliber and fibrous cap formation during atheroma development in mice.[9] In advanced lesions, atherosclerosis-prone mice with disrupted IL-1 signaling developed significantly more luminal encroachment, attributed to an apparent defect in expansive remodeling that generally accompanies the growth of atherosclerotic plaques.[10] Reduced matrix metalloproteinase-3 (MMP-3) expression by vascular smooth muscle cells (VSMCs) in atheromata of mice with defective IL-1 signaling presumably impaired the remodeling of the extracellular matrix required for the outward expansion of arteries as well as VSMC migration into the intima, a process implicated in the formation of a fibrous cap. These results suggest that IL-1 promotes “compensatory” expansive remodeling of atherosclerotic arteries and favors fibrous cap formation.

Many enzymes likely participate in vascular remodeling, and the functions of specific proteinases may differ substantially between humans and mice.[11] We and others have localized MMP-3(6) or its messenger RNA[12] in human atherosclerotic plaques. Schoenhagen et al.[13] studied atheromatous tissue of patients subjected to percutaneous intervention and found that greater expression of MMP-3—but not of MMP-1—in atheromata accompanied expansive remodeling of the arterial segment.

The defect in compensatory remodeling observed in atherosclerotic IL-1R1-deficient mice[9, 14] raised concerns[14] about possible limitations of blocking the IL-1 receptor as a therapeutic approach for cardiovascular disease.[15–20] The selective neutralization of the β isoform of IL-1, currently being tested in CANTOS, a large Phase 3 outcomes trial in humans with atherosclerosis, offers an alternative approach to blockade of both IL-1 isoforms, for example with the IL-1 receptor antagonist (IL-1Ra, anakinra).[21, 22] Rigorous definition of the contributions of the IL-1 isoforms to mechanisms related to arterial remodeling in human cells therefore not only has considerable mechanistic interest but also assumes critical clinical importance.

This study investigated whether IL-1α and IL-1β overlap in regulating the expression of MMP-3 and other remodeling-related proteases in isolated human cells relevant to atherosclerosis in primary culture and in fresh human carotid endarterectomy specimens. Our results demonstrate that either IL-1α or IL-1β similarly induce the expression of proteases by human VSMCs *in vitro* and by human atheromatous tissue *ex vivo*. The results further showed that the expression of IL-1α and IL-1β correlates with MMP-3 expression in atheromata. Demonstration of the redundancy of IL-1 isoforms in human cells furnishes new insight into the mechanisms of arterial remodeling, and also provides important information regarding the selective targeting of IL-1 isoforms in the chronic treatment of atherosclerosis and other cardiovascular diseases.

## Materials and Methods

### Cell and *Ex-Vivo* Tissue Cultures

Tissue specimens discarded after carotid endarterectomy surgery were cleaned by rinsing 3 times with sterile Hank’s buffered saline solution, sliced into cross-sectional segments approximately 2–3 mm wide, and placed in separate wells of a 24-well plate with 0.5 to 1 ml of Dulbecco’s modified Eagle medium (DMEM) supplemented with insulin, transferrin, and selenium (ITS, Gibco, Life Technologies),[23] as well as penicillin, streptomycin, and amphotericin. Alternate sections were incubated in the presence of 1040 pM IL-1α or IL-1β, (PeproTech, Rocky Hill, NJ), or left unstimulated for 48 hours. MMP-3 concentration in conditioned media was determined by enzyme-linked immunosorbent assay (ELISA) and the values were normalized to total tissue protein content, as assessed by the BCA assay (Thermo Scientific).

Endothelial (HSVECs) and VSMCs from saphenous vein segments discarded from coronary artery bypass surgeries were obtained as described previously.[24, 25] Peripheral blood monocytes were isolated from buffy coats from consenting, healthy donors and differentiated to macrophages as described.[26] Persuant to Protocol 1999P001348, the Partners Human Research Committee approved a waiver of informed consent and authorization for the use of excess human material from clinical procedures that would otherwise be discarded as allowed by and in compliance with federal regulations 45 CFR 46.116 and the Privacy Rule. The review of this research meets the requirements in the Declaration of Helsinki and this study was approved Partners Human Research Committee. The Partners Human Research Committee is responsible for review and oversight of research conducted by researchers at Brigham and Women’s Hospital, Massachusetts General Hospital, McLean Hospital, and North Shore Medical Center.

VSMCs (passage 3 to 6) were cultured to 90% confluence in DMEM containing 10% fetal bovine serum (FBS). After incubation for 24 hours in serum-free DMEM containing ITS,[27] cells were stimulated with up to 570 pM of IL-1α or IL-1β, or left unstimulated for 6–8, 24, and 48h. Conditioned media and cell lysates were harvested for mRNA and protein expression analyses. To perform experiments with HSVECs or macrophages, cultures were incubated overnight in a step-down medium (M199 containing 5% FBS for HSVECs or RPMI 1640 supplemented with 1% human serum for macrophages), and then cells were stimulated using the same protocol as for VSMCs. These experiments used 10 ng/ml of IL-1 isoforms, a concentration widely used for cytokine studies (570 pM corresponds to 10.26 ng/ml of IL-1α, and to 9.86ng/ml of IL-1β). To improve tissue delivery, *ex-vivo* specimen culture experiments used twice these concentrations.

### RNA Extraction and Reverse Transcription-Quantitative Polymerase Chain Reaction (RT-qPCR)

We extracted RNA with RNeasy columns (Qiagen, Valencia, CA) and synthesized cDNA with SuperScript First-Strand (Invitrogen, Carlsbad, CA). For RT-qPCR, we used the following pairs of primers: 18S (endogenous control, NCBI Gene ID 106632259): 5’-ATGGCCGTTCTTAGTTGGTG-3’ and 5’- GAACGCCACTTGTCCCTCTA-3’; MMP-3 (NCBI Gene ID 4314): 5’- CAAAACATATTTCTTTGTAGAGGACAA-3’ and 5’- TTCAGCTATTTGCTTGGGAAA-3’; IL-6 (NCBI Gene ID 3569): 5’- AGTGAGGAACAAGCCAGAGCTGTCC-3’ and 5’- AATCTGAGGTGCCCATGCTACATTTG-3’; vascular cell adhesion molecule 1 (VCAM-1, NCBI Gene ID 7412): 5’-AAGATGGTCGTGATCCTTGG-3’ and 5’- GGTGCTGCAAGTCAATGAGA-3’; MMP-1 (NCBI Gene ID 4312): 5’- ACACATCTGACCTACAGGATTGA-3’ and 5’- GTGTGACATTACTCCAGAGTTGG-3’; MMP-8 (NCBI Gene ID 4317): 5’- TCTTTGTAAATGACCAATTCTGGA-3’ and 5’-GGAAAGGCACCTGATATGCT-3’; MMP-12 (NCBI Gene ID 4321): 5’-TGTCACTACCGTGGGAAATAAG-3’ and 5’- AACACTGGTCTTTGGTCTCTCAG-3’; cathepsin S (NCBI Gene ID 1520): 5’- CGACGTCTCATCTGGGAAA-3’ and 5’-AAGACATCACTTCTTCACTGGTCA-3’. Relative gene expression was calculated with the ΔΔCt method.

### ELISA, Immunoblot and Zymography

Total MMP-3 in cell-conditioned media was quantified by ELISA according to the manufacturer’s instructions (R&D Systems). For immunoblotting, samples were fractionated on 4%–12% gradient SDS-PAGE gels (Life Technologies, Grand Island, NY) and transferred to polyvinylidene difluoride membranes. After blocking with 5% defatted milk and incubating with the appropriate antibodies, membranes were developed using a chemiluminescence reagent (Thermo Scientific, Waltham, MA). VSMC- conditioned media was concentrated 10-fold with centrifugal filter units (Millipore, Billerica, MA) prior to electrophoresis. The following antibodies were used: anti-MMP-3 (1mg/ml, ab38907, Abcam, Cambridge, MA), anti-MMP-1 (0.2mg/ml, sc-8834-R, Santa Cruz, Santa Cruz, CA), anti-MMP-8 (0.1 mg/ml, AF908, R&D Systems, Minneapolis, MN), anti-MMP-12 (1 mg/ml, MAB919, R&D Systems), and anti-Cathepsin S (0.2 mg/ml, ab18822, Abcam).

MMP-2 and MMP-9 activities were assessed in conditioned media by gelatin zymography. Samples were fractionated under non-reducing conditions on 10% Tris-glycine gels containing 0.1% gelatin substrate (Novex, Life Technologies) and renatured for 1 hour at room temperature in Novex Renaturing buffer, incubated for 24 hours at 36°C in Novex Developing buffer, and stained with SimpleBlue SafeStain (Life Technologies). Densitometric analyses of immunoblots and zymography gels were performed using Image J (National Institutes of Health). For *ex-vivo* tissue culture samples, all results were normalized to the total protein content in tissue sections, as assessed by the BCA assay.

### MMP enzymatic activity assay

MMP activity was measured with MMP-3 Green substrate (ab112148, Abcam, MA), a fluorogenic peptide more selective for MMP-3 than for other MMPs under the reaction conditions recommended by the manufacturer. Conditioned media were concentrated 7-fold with Centrifugal Filter Units (UFC503096, Millipore, USA) and incubated with the substrate for 2 hours at 37°C. The fluorescence signal was monitored at Ex/Em = 485/538nm in a Spectramax M2 (Molecular Devices, USA), subtracting the background fluorescence from all readings.

### Cell migration assay

Migration assays were performed in modified Boyden chambers, using cell culture inserts with 8-μm pore membranes coated with Matrigel in 24-well plates. VSMCs (1x104 cells) were seeded on top of polymerized Matrigel (1mg/ml, 20 μl per insert). After cell attachment and incubation in serum-free medium for 24 hours, 10 ng/ml IL-1α or IL- 1β was added to the lower and the upper chambers (no gradient), incubated for 24 hours, and then human recombinant platelet-derived growth factor-BB (PDGF-BB, GenWay Biotech, San Diego, CA) was added to the lower chamber at a concentration of 10 ng/ml. After allowing cells to migrate for 24 hours, they were fixed in methanol, stained with HEMA-3 stain set, and counted in 6 fields at 20x magnification.

### Immunohistochemistry

Human carotid endarterectomy specimens embedded in Optimal Cutting Temperature medium were frozen on dry ice and stored at -80°C. Serial cryostat sections (6 μm) were cut, air-dried onto microscope slides (Fisher Scientific, Pittsburgh, PA), and fixed in acetone at -20°C for 5 min. After pre-incubating sections with phosphate buffered saline (PBS) containing 0.3% hydrogen peroxide to suppress endogenous peroxidase activity and with protein block (serum free, ready-to-use, No X0909; DAKO) + 1% BSA, the sections were incubated with primary antibodies diluted in PBS supplemented with 5% appropriate serum overnight at 4°C. The following primary antibodies were used: goat anti-human MMP-3 (1:20, AF513, R&D Systems), mouse anti-human IL-1α (1:50, MAB200, R&D Systems), rabbit anti-human IL-1β (1:60, sc-7884, Santa Cruz), mouse anti-human α-smooth muscle actin (α-SMA) (1:35, ENZ-C34931, Enzo Diagnostics, Syosset, NY), and mouse anti-human CD68 (1:700, M071801-8, Dako Co, Carpinteria, CA). The subsequent processing was performed according to the manufacturer’s recommendations (Universal Dako LSAB kit, peroxidase, K0675; DAKO Co); sections were incubated with biotin-conjugated secondary antibodies for 30 minutes and with streptavidin for 20 minutes at room temperature while washing with PBS between steps (3X5 minutes). Antibody binding was visualized with 3-amino-9-ethyl carbazole (ready-to-use, K3464: DAKO). Mouse IgG, rabbit IgG, or goat IgG, were used as negative controls to confirm antibody specificity. Sections were counterstained with Gill’s hematoxylin solution (Sigma) and mounted using water-soluble mounting media. The histologic sections were photographed with 100x magnification and analyzed with Image Pro image analysis software (Media Cybernetics, Rockville, MD). All measurements were performed after spatial calibration. Tunica intima and tunica media were measured separately and stainings quantified independently. Results are reported as percentage of area positive for staining.

### Statistical Analyses

Results are reported as mean ± standard error of the mean (SEM). When 3 groups were compared, ANOVA, Friedman (paired), or Kruskall-Wallis (unpaired) tests with appropriate post-hoc testing (Tuckey’s or Dunn’s test) for comparison between groups were utilized. Linear regression tested the association between percentage area positive for MMP-3 and the markers examined in the immunohistochemistry studies. Multivariate analysis was conducted with principal component regression[28] using IL-1α, IL-1β, CD68, and SMA as independent variables and MMP-3 as the dependent variable. The results were reported as β coefficients ± 95% confidence intervals and a linear regression for MMP-3 predicted by the multivariate model vs. MMP-3 observed in the samples, representing fitness of the model. The R2 for the later regression equals the R2 for the multivariate model.

## Results

**Fig 1:**
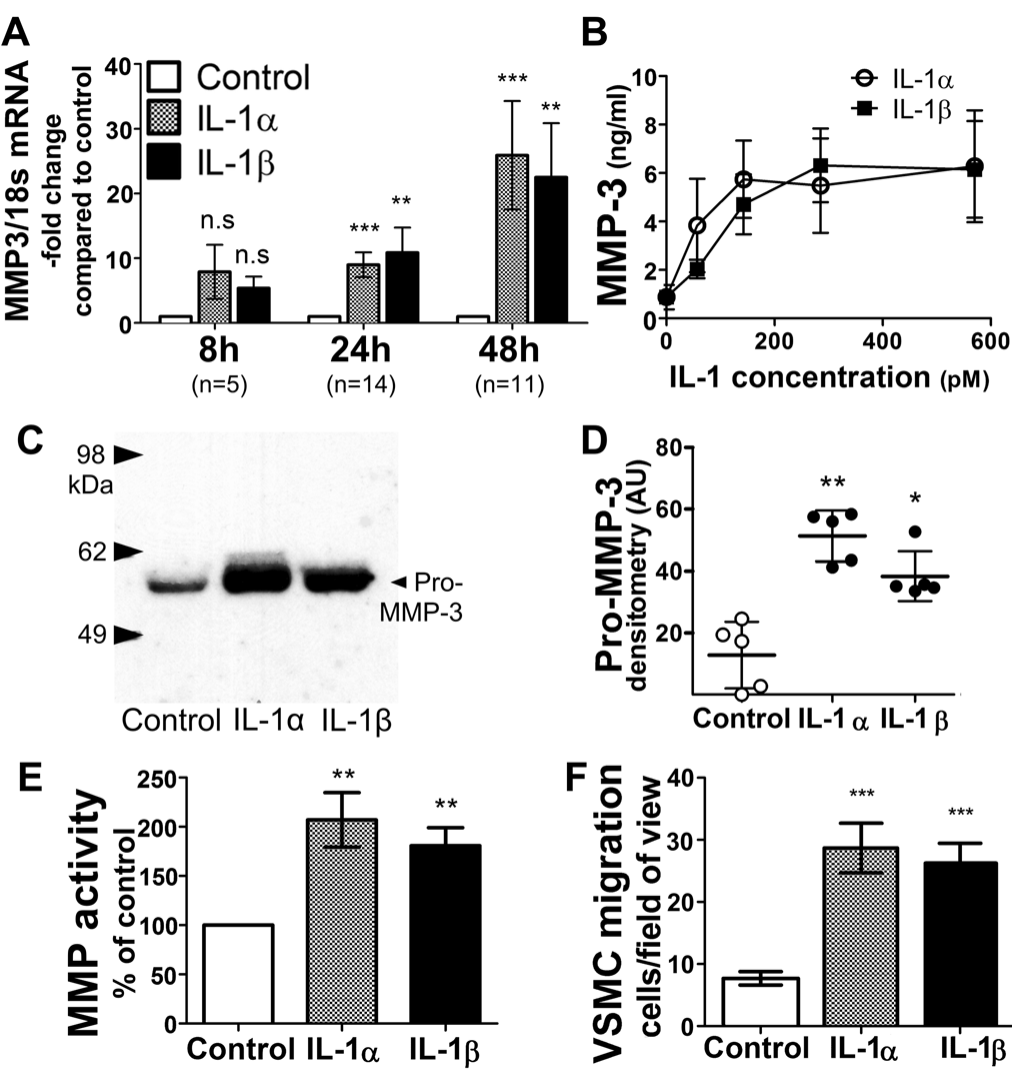
IL-1α and IL-1β induce similar levels of MMP-3 mRNA and protein expression in VSMCs. (A) Cells were incubated with 570 pM IL-1α or IL-1β for the indicated periods of time (n = 5, 14, and 11), followed by mRNA extraction and determination of MMP-3 mRNA levels by RT-qPCR. Levels of 18S RNA served as an internal control for adjustment between samples (P values **<0.01, ***<0.001, n.s. = not significant compared to control). (B) Cells were stimulated with various concentrations of IL-1α or IL-1β for 48 hours, and the MMP-3 concentration in cell supernatants was determined by ELISA (n = 4–6, responses to IL-1 isoforms did not differ significantly at any concentration tested). (C) Cells were stimulated by 570 pM IL-1α or IL-1β for 48 hours and the supernatants were analyzed by immunoblot. (D) Pooled data from densitometric analysis of immunoblots (n = 5, Tuckey’s post hoc test P values *<0.05, **<0.01). (E) MMP activity secreted by IL-1–treated VSMCs. Conditioned media from VSMCs stimulated with 570 pM of either IL-1 isoform for 48h were incubated with MMP Green substrate for 2 hours and fluorescence was measured at Ex/Em = 485/538nm. Background fluorescence was subtracted from all readings. (n = 4–6, Dunn’s post hoc test P value **<0.01). (F) VSMC migration towards PDGF-BB through Matrigel. Pooled data from 5 independent experiments are presented as mean ± SEM, ***P<0.001 for Dunn’s post hoc test. P values are indicated in all panels as *<0.05, **<0.01, ***<0.001 compared to control; n.s. = not significant for Tuckey’s test.

The initial incubation of cultures of primary human cells relevant to atherosclerosis with equimolar concentrations of each IL-1 isoform, and the examination of MMP-3 mRNA expression, protein secretion, and activity helped to assess rigorously the potential functional overlap of IL-1α and IL-1β. Treatment of VSMCs with either IL-1 isoform induced similar amounts of MMP-3 mRNA expression (Fig 1A) and pro- MMP-3 protein secretion (Fig 1C) compared to untreated cells, as assessed by RT-qPCR or immunoblot, respectively. IL-1-induced elevation of MMP-3 mRNA levels remained modest at shorter time intervals (8 hours) and more marked at longer incubation times (24 and 48 hours), suggesting autocrine or paracrine action of cytokines released early in the incubation (Fig 1A). MMP-3 secretion depended similarly on the concentration of either cytokine, as determined by ELISA (Fig 1B). Stimulation of VSMCs with either IL-1 isoform increased secreted MMP activity, assessed with a fluorogenic peptide cleaved preferentially by MMP-3 (Fig 1E).

Cell migration, a process crucial to vascular remodeling, relies on the coordinated expression of many proteases and other proteins.[29, 30] Alexander et al.[9] described features of plaque instability in mice deficient in IL-1R1, and attributed part of those findings to the failure of IL-1-induced, MMP-3-dependent VSMC migration into the plaque and subsequent formation of fibrous cap. A modified Boyden chamber migration assay through Matrigel®, a basement membrane–type mixture of extracellular matrix constituents, helped test the hypothesis that either IL-1α or IL-1β can facilitate VSMC migration.[9] VSMCs seeded over a polymerized Matrigel barrier on the upper chamber and exposed to either IL-1 isoform or no cytokines for 48h underwent stimulation of cell migration by the addition of the chemoattractant PDGF-BB to the lower compartment. Treatment of cells with either IL-1α or IL-1β elicited similar increases in PDGF-BB–induced cell migration compared to cells not treated with either cytokine (Fig 1F), indicating functional redundancy of both IL-1 isoforms.

In contrast to their effect on VSMCs, IL-1 isoforms did not increase MMP-3 mRNA expression or protein secretion in macrophages or HSVECs (Data A in S1 Dataset). The MMP-3 concentration in media conditioned by control or IL-1-stimulated HSVECs in three independent experiments remained undetectable by ELISA, while secreted MMP-3 remained barely detectable in one out of three independent experiments in macrophages, and did not increase with IL-1 treatment (Data B in S1 Dataset). Under the same experimental conditions, IL-1 isoforms induced robust expression of the internal positive control IL-6 in macrophages and vascular cell adhesion molecule-1 (VCAM-1) in HSVECs (Data A in S1 Dataset).

**Fig 2:**
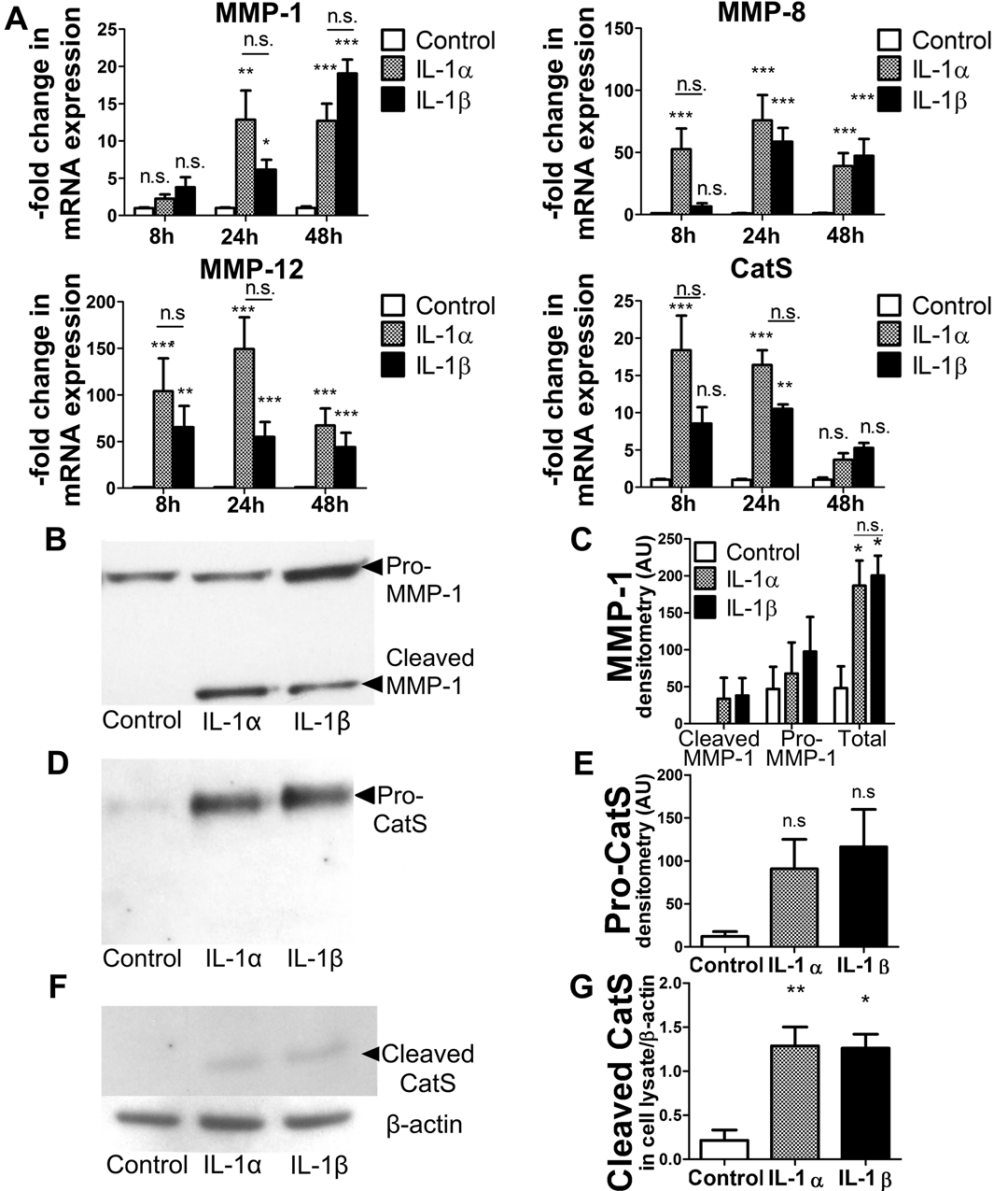
IL-1α and IL-1β similarly augment expression of several matrix-degrading enzymes and facilitate cell migration in VSMCs. (A) Cells were incubated with 570 pM IL-1α or IL-1β for the indicated periods of time, followed by mRNA extraction and determination of mRNA levels by RT-qPCR (n = 11, 14, and 11 for 8, 24, and 48h, respectively). Amounts of 18S RNA served as an internal control for adjustment between samples. No statistically significant difference occurred between IL-1 isoforms at any time point. (B-G) Cells were stimulated by 570 pM IL-1α or IL-1β for 48 hours and the supernatants (B and D) or cell lysates (F) were analyzed by immunoblot. C, E, and G panels show densitometric analyses of the respective immunoblots (C, n = 4; E, n = 3; G, n = 3) Data from densitometric analyses are presented as mean ± SEM.

Arterial remodeling involves the degradation of multiple components of the extracellular matrix including collagen types I and III and elastin, poor substrates for MMP-3. IL-1 can induce VSMCs to secrete additional proteases that may contribute to matrix remodeling.[8, 25] This study therefore involved screening VSMCs stimulated with IL-1 isoforms for the induction of proteases previously implicated in atherosclerosis. Either IL-1 isoform similarly increased mRNA expression of MMP-1, MMP-8, MMP-12, and cathepsin S (CatS) (Fig 2A), and did not modify concentrations of mRNA encoding MMPs-2, -7, -9, -13, -14 or cathepsins K or L (not shown). Treatment with either IL-1 isoform elicited secretion of similar amounts of pro- and cleaved MMP-1 (Fig 2B and 2C) and pro-CatS (Fig 2D and 2E), and induced comparable levels of intracellular expression of cleaved CatS (Fig 2F and 2G). Immunoblots did not detect MMP-8 or MMP-12. Collectively, these results indicate that IL-1α or IL-1β induce a similar set of matrix-degrading proteases in VSMCs.

**Fig 3:**
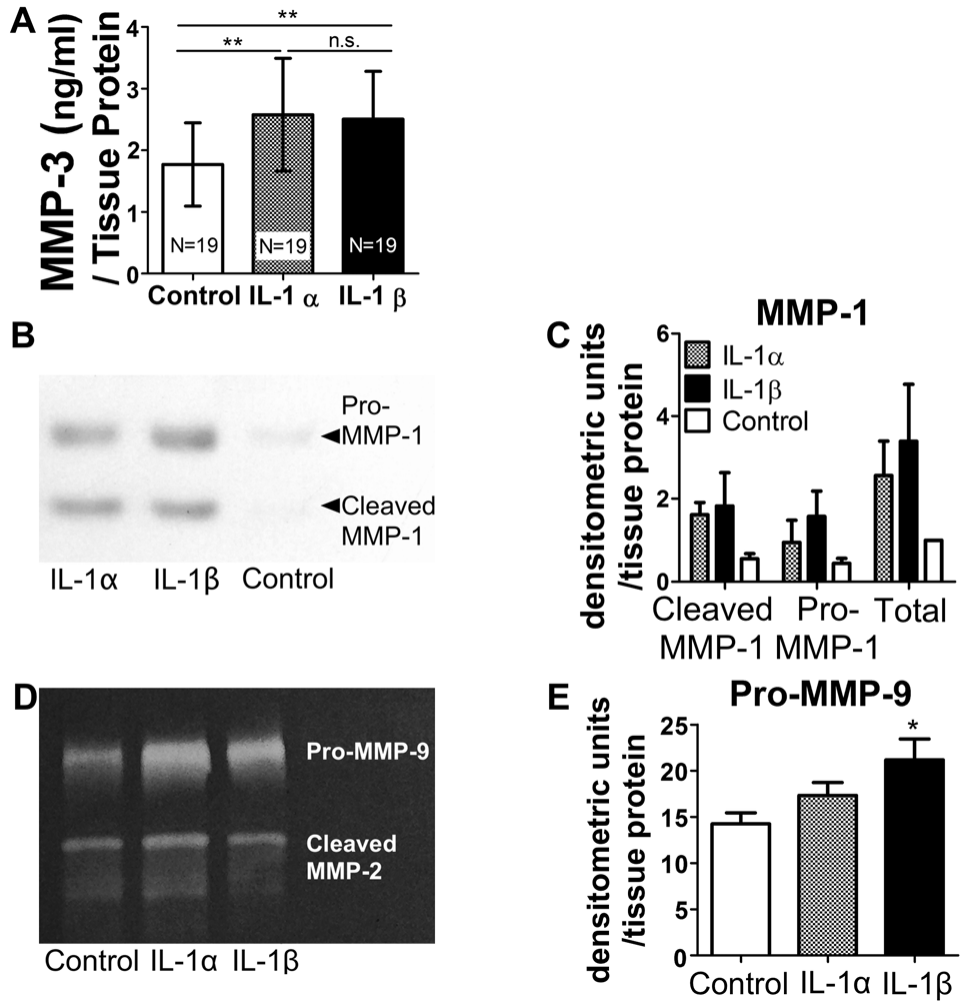
IL-1α and IL-1β elicit similar levels of protease expression in *ex-vivo* tissue cultures of human atheromata. Cultures were stimulated by 1040 pM IL-1α or IL-1β for 48 hours and the levels of the indicated proteases were quantified in conditioned media. The results are normalized to protein concentration in tissue lysates. (A) MMP-3, determined by ELISA. The groups (n = 19) were compared with Friedman’s test and Dunn’s post hoc test; **P<0.01, n.s., not significant. (B) Immunoblotting of MMP-1 containing a 53kDa band corresponding to the pro-enzyme and a 45kDa band corresponding to the cleaved form. (C) Normalized densitometric units of MMP-1 immunoblots (n = 3, groups did not differ significantly). (D) Gelatin zymography demonstrating gelatinolytic activity corresponding to pro-MMP9 at 92kDa and cleaved MMP-2 at 63kDa. (E) Normalized densitometric analyses of zymograms (n = 7, *P<0.05).

The present study also examined the effect of equimolar concentrations of IL-1 isoforms on protease secretion in *ex-vivo* tissue cultures of fresh human carotid endarterectomy specimens. Given the inherent heterogeneity of these specimens, multiple alternate adjacent slices underwent exposure to the various conditions, as previously described.[26, 31] Similar to their effect on isolated VSMCs, IL-1α or IL-1β induced comparable increases in secretion of MMP-3 as assessed by ELISA (Fig 3A). IL-1 treatment elicited increases in pro-, cleaved, and total MMP-1 secretion, evaluated by immunoblot in three groups of carotid sections (Fig 3B). Pooled differences between the treated samples and controls did not reach statistical significance (Fig 3C), likely due to the variability in the magnitude of response in different donors. Additionally, treatment with IL-1β augmented MMP-9 activity in culture supernatants, as determined by gelatin zymography. Stimulation with IL-1α showed a similar trend, but the results did not reach statistical significance (Fig 3D and 3E). Conditioned media did not contain CatS under these conditions. Taken together, these results indicate that human atheroma, a tissue of much higher complexity than cell monolayers, responds to stimulation with either IL-1 isoform by augmenting the expression of matrix-degrading proteases. The relatively high baseline expression of these enzymes likely reflects the tonic inflammatory milieu of these atheroma specimens selected for surgical intervention.

**Fig 4:**
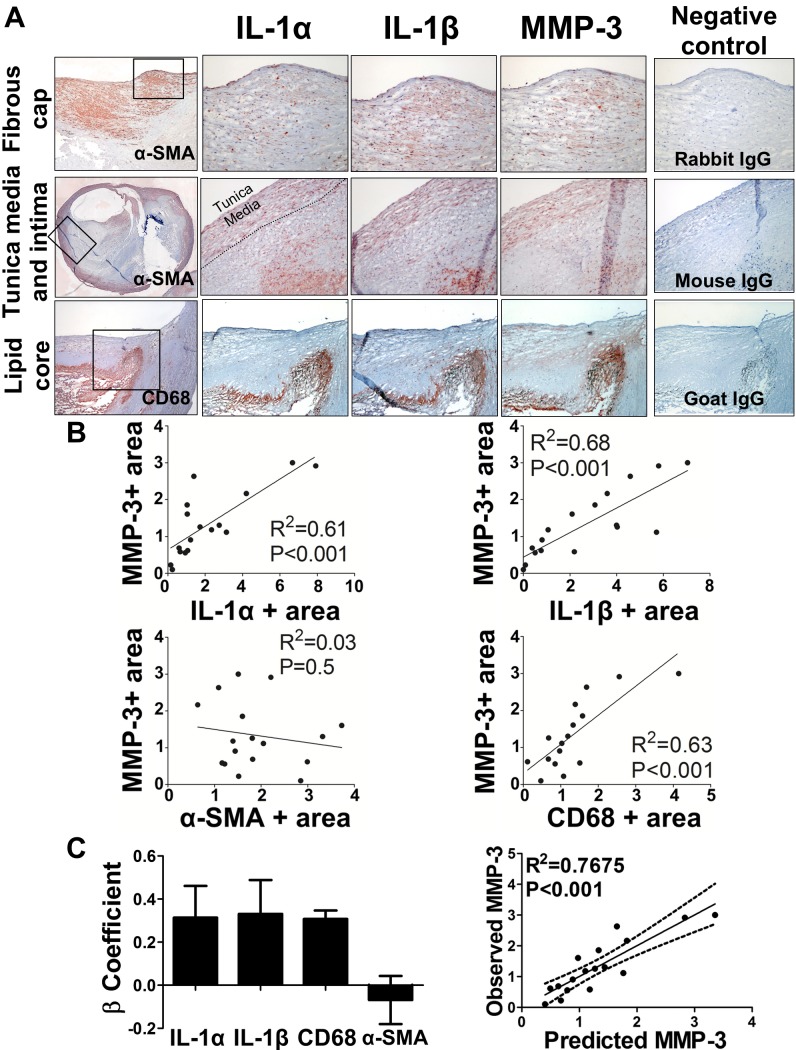
The expression of MMP-3 associates with the expression of IL-1α and IL-1β in human atheroma. (A) Representative immunohistochemical stainings of IL-1 isoforms, MMP-3, SMC α-actin (α-SMA), and CD68 in fibrous cap (top panels, original magnification 100x); tunica media and tunica intima (middle panels, 100x); and macrophage-rich shoulder area with lipid core (bottom panels). On the first column, black rectangles designate areas magnified on the following panels. Controls for antibody specificity using rabbit, mouse, or goat IgG were performed on serial sections (right panels) (B) Linear regressions demonstrate the relationship between MMP-3^+^ area and IL-1α (upper left), IL-1β (upper right), αSMA (lower left), and CD68^+^ (lower right) areas in the tunica intima (n = 17 for MMP-3, IL-1α and IL1-β; n = 16 for CD68). (C) A principal component regression demonstrates that IL-1α, IL-1β, and CD68^+^ areas associate similarly and significantly with the MMP-3^+^ area. Left panel: bar graphs represent β-coefficient and 95% confidence intervals. Right panel: regression of predicted vs. observed MMP-3^+^ area demonstrates fitness of the model and global R^2^ = 0.77. Dashed lines represent 95% confidence intervals.

Further experiments tested the hypothesis that the presence of either IL-1 isoform would associate with the expression of MMP-3 in human atheromata, a scenario consistent with their redundant function in lesion’s expansive remodeling. Both IL-1 isoforms and MMP-3 localize to similar areas in the fibrous cap, tunica media, tunica intima, and macrophage-rich shoulder areas of human carotid endarterectomy specimens, as assessed by immunohistochemistry (Fig 4A). Lipid core areas of lesions barely showed immunodetectable IL-1 isoforms and MMP-3. Quantitative analysis of the immunostaining images revealed that the percentages of IL-1α+ and IL-1β+ areas in the intima associate with the MMP-3+ area (R2 = 0.61 and 0.68, respectively) (Fig 4B, upper panels), thus supporting the hypothesis that both IL-1 isoforms contribute to MMP-3 expression in atheromata.[30] CD68+ area, but not α -SMA+ area, also correlated positively with MMP-3 (Fig 4B, lower panels). Multivariate analysis confirmed that a similar magnitude of the association, given by the β coefficient, existed for the 3 variables (Fig 4C left panel). The regression for predicted MMP-3 vs. observed MMP-3, which represents the fitness of the model (R2 = 0.77, Fig 4C right panel), demonstrates that IL-1α, IL-1β, and CD68+ cells accounted for 77% of the variance of MMP-3 observed in human endarterectomy species. These data further support the hypothesis that the expression of MMP-3 largely associates with the presence of both IL-1 isofoms in the tunica intima.

## Discussion

A large body of experimental data has implicated IL-1 isoforms in atherogenesis.[32] Hence, IL-1 has emerged as a therapeutic target in patients with or at risk for atherosclerosis.[15] One approach to limiting IL-1 signaling *in-vivo* uses IL-1Ra, a naturally occurring member of the IL-1 family that blocks the IL-1 type 1 receptor (IL-1R1), which transduces signals from both isoforms. Yet, a study in mice with genetic disruption of IL-1R1 signaling unveiled a potential limitation to this approach.[9] In the work by Alexander et al.,[9] the failure of compensatory expansive remodeling in *Il1r1*^*-/-*^ mice with advanced atherosclerosis prompted a search for a putative mechanism. Among all cell types involved in atherosclerosis, their investigation focused on vascular smooth muscle cells (VSMCs) for two reasons: 1) expansive remodeling involves the reorganization of the muscular layer of arteries, and 2) atheromata of *Il1r1*^*-/-*^ mice contained fewer alpha-SMA+ cells than controls, indicating that the abrogation of IL-1 receptor signaling limited VSMC migration into the intima. This approach led to the identification of reduced expression of MMP-3 by VSMCs as a potential mechanism of decreased expansive remodeling in *Il1r1*^*-/-*^ mice. This finding, together with our interest in elucidating the role of IL-1 isoforms in compensatory vascular remodeling, prompted a head-to-head comparison of the effects of each IL-1 isoform in human cells relevant to atherosclerosis and in *ex-vivo* in human carotid endarterectomy specimens, and complemented the study with examination of the expression of IL-1 isoforms and MMP-3 in human atheromata. The results affirm in human VSMCs the effect of IL-1 isoforms on MMP-3 expression observed in mice, and advance the observations regarding IL-1-induced VSMC migration. In an apparent contradiction with the results obtained with cultured cells, the immunohistochemistry studies of human carotid endarterectomy specimens revealed no correlation between MMP-3 and αSMA+ cell content within lesions. This discrepancy may result from a limitation from a selection bias inherent in study of operative specimens of carotid plaques, since patients undergoing endarterectomy frequently harbor advanced, highly inflamed atherosclerotic lesions skewed toward a high macrophage content relative to smooth muscle cells.

Our results showed a positive association between the expression of IL-1 isoforms and MMP-3 in macrophage-rich regions of atheromata although IL-1 does not induce MMP-3 expression in cultured macrophages. These observations support the view that elaboration of MMP-3 in human atheromata likely stems from the concerted action of cytokines and damage-associated molecules released from dead or dying cells rather than from the direct and sole action of IL-1 isoforms on macrophages. This finding in human tissues differs with the findings of mice of Alexander et al[9] that advanced, macrophage-rich atheromata from *Il1r1*^-/-^ animals contained virtually no MMP-3 detectable by immunohistochemistry. The present study’s conclusion highlights the risk of extrapolating results obtained using germline deletion of IL-1 receptor in mice—which may profoundly influence the biology of monocytes and macrophages—to human atherosclerosis, a complex and multifactorial disease. In any case, these data support our hypothesis that pharmacological neutralization of a single IL-1 isoform in humans will not reduce the expression of MMP-3 and other remodeling-relevant proteases to the same extent as in *Il1r1*^-/-^ mice, making it unlikely for this intervention to cause a significant encroachment of arterial lumen.

Mice and humans have a distinct repertoire of matrix-degrading proteinases. For example, mice lack MMP-1, one key interstitial collagenase in humans. Also, elastolysis in human arteries may depend more on cysteinyl proteinases than on metallo-elastases. This spurred the investigation of the effect of IL-1 isoforms on the expression of other remodeling-relevant proteases. Our results indicate that IL-1α and IL-1β induce similar amounts of MMP-3 and MMP-1 in human carotid endarterectomy specimens *ex-vivo* and comparable levels of expression of these MMPs and of CatS in human VSMCs *in-vitro*. Additionally, IL-1β increased MMP-9 activity in human atheromatous tissue *ex-vivo*, and IL-1α showed a similar trend. Incubation with either IL-1 isoform also increased migration of human VSMCs induced by PDGF to similar extents. Therefore, the action of IL-1α may suffice to allow cell migration and compensatory remodeling when targeting IL-1β to reduce inflammation in atherosclerosis, avoiding the potentially adverse effects of abrogation of signaling by both isoforms noted in IL-1R1-deficient mice.[9]

Collectively, our data demonstrate that IL-1α and IL-1β can exert redundant effects *in vitro* and colocalize in human atheroma, where they may participate in processes leading to local protease secretion. The selective neutralization of one of the IL-1 isoforms will therefore likely reduce but not abolish the expression of proteases implicated in vascular remodeling. Ultimate evidence regarding the efficacy and safety of IL-1β neutralization in humans with established atherosclerosis will emerge from sufficiently powered clinical endpoint trials such as CANTOS. The data from the present study not only provide novel mechanistic information about the regulation in human cells and atheromata of matrix- remodeling enzymes implicated in atheroma complications, but will also inform the interpretation of the results of ongoing clinical trials.

## Supporting Information

S1 Dataset Supplemental data and main results dataset. (XLS)

S1 Fig Western blots of vascular smooth muscle cell culture. (PDF)

S2 Fig Western blots and zymograms of ex-vivo tissue culture. (PDF)
