# Supplementary material for: Redundancy of IL-1 Isoform Signaling and Its Implications for Arterial Remodeling
Source: PLoS One. 2016 Mar 31;11(3):e0152474. doi: 10.1371/journal.pone.0152474 (PMC4816548; doi:10.1371/journal.pone.0152474)
Supplement: S1 Fig — (PDF) [file pone.0152474.s002.pdf]

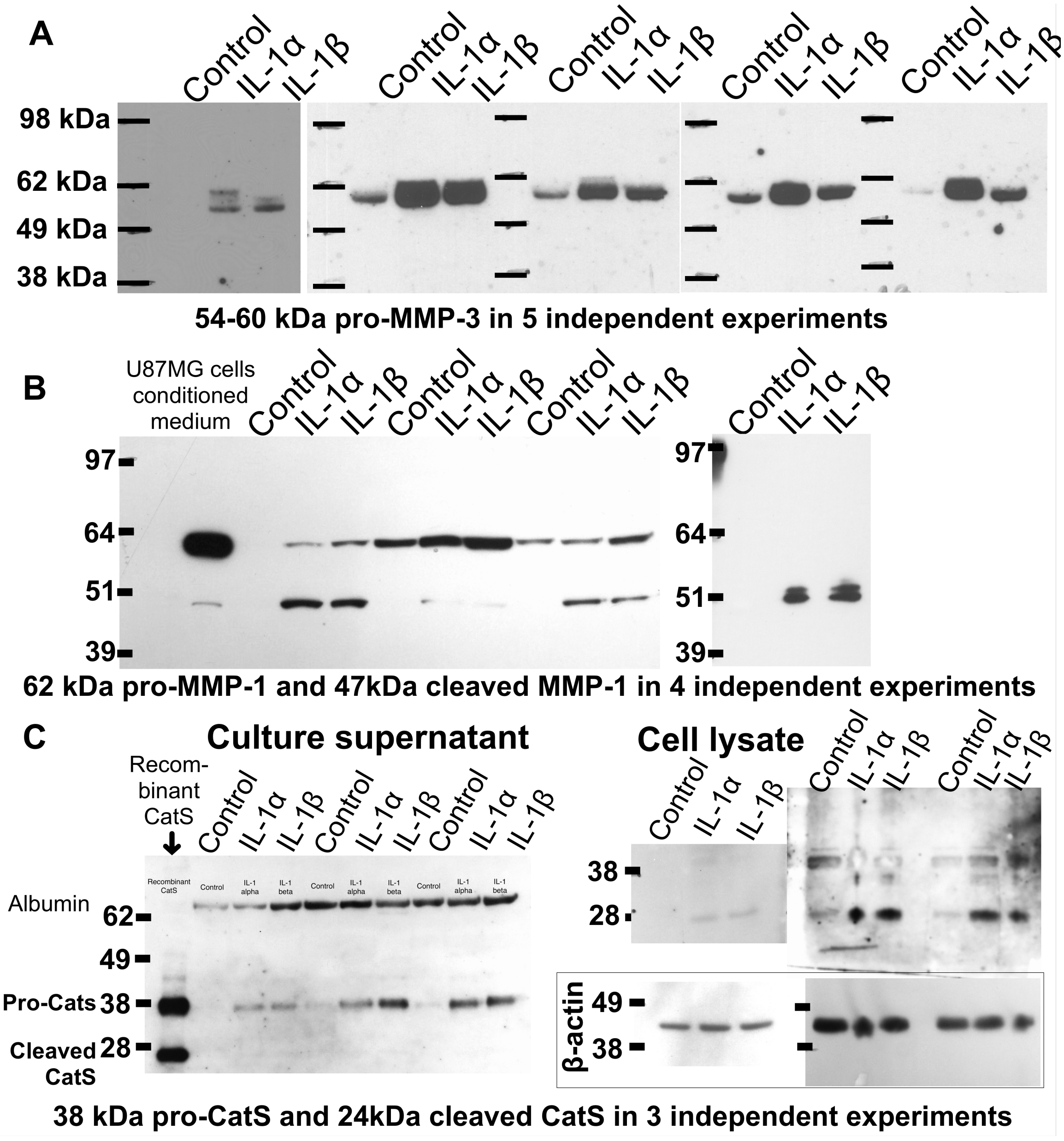

S1 Figure. VSMCs express proteinases after stimulation with either IL-1 isoform. (A) Five independent experiments revealed pro-MMP-3 in VSMCs-conditioned media. (B) In three experiments, conditioned media contained the pro- and cleaved forms of MMP-1; in one experiment only cleaved MMP-1 was detected. (C) Culture supernatants (left) and cell lysates (right) of VSMCs contain Cathepsin S (CatS). Conditioned media presented only the pro-form of CatS after

IL-1 stimulation. Both pro- and cleaved CatS were detected in cell lysates, but only cleaved CatS significantly increased following IL-1 stimulation. Albumin in serum-containing medium was detected as band at 65kDa due to antibody nonspecific binding. β-actin served as loading control for cell lysate samples (box on bottom right).
