# Supplementary material for: Redundancy of IL-1 Isoform Signaling and Its Implications for Arterial Remodeling
Source: PLoS One. 2016 Mar 31;11(3):e0152474. doi: 10.1371/journal.pone.0152474 (PMC4816548; doi:10.1371/journal.pone.0152474)
Supplement: S2 Fig — (PDF) [file pone.0152474.s003.pdf]

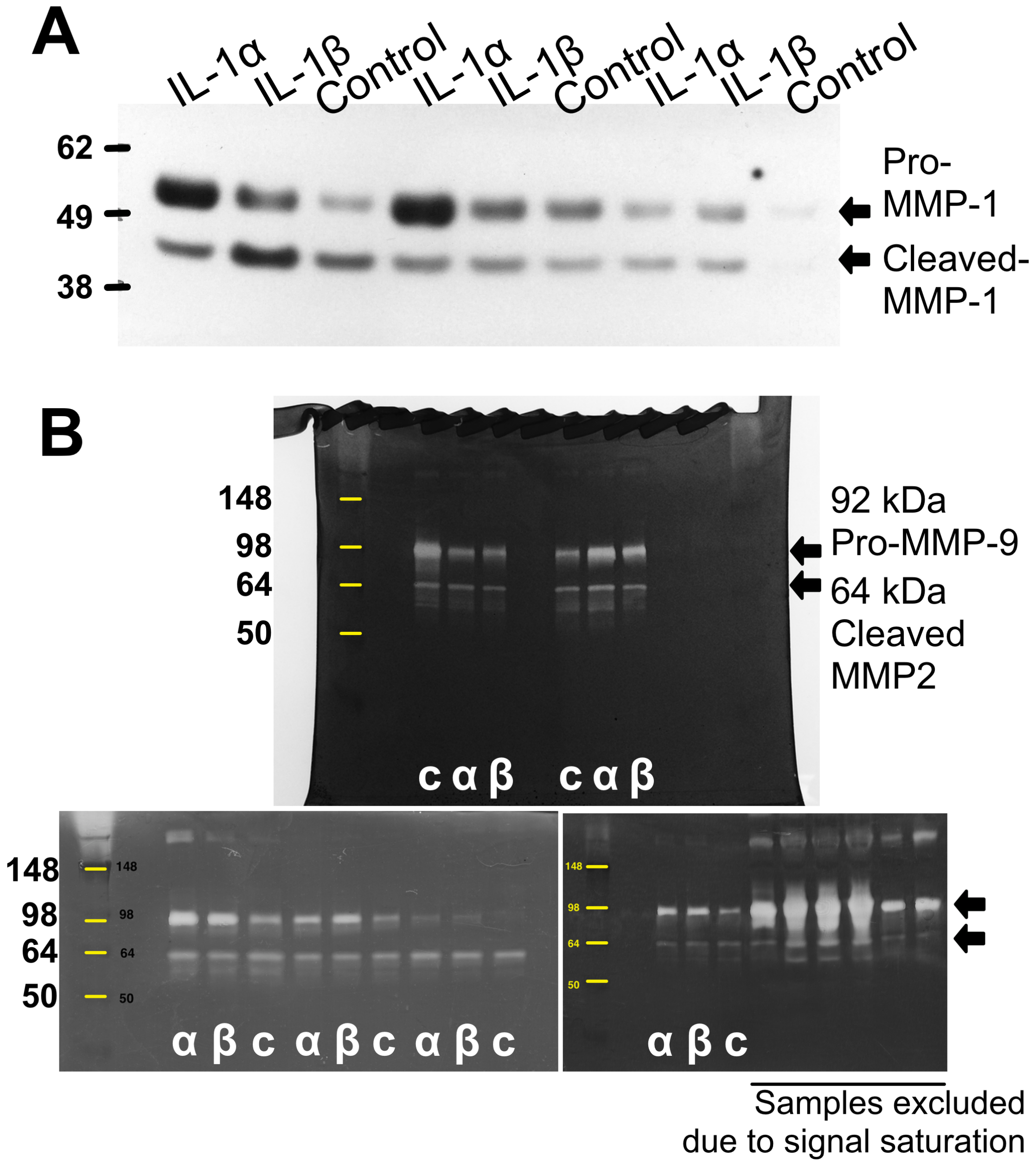

S2 Figure. **Ex vivo carotid tissue increases proteinase expression after stimulation with either IL-1 isoform.** (A) Western blot demonstrates the presence of pro- and cleaved MMP-1 in ex-vivo tissue culture-conditioned media. Densitometric analyses of the three experiments shown were further corrected according to total protein extracted from tissue. (B) Gelatin zymography of ex-

vivo tissue culture -onditioned media demonstrates that IL-1 isoforms tend to up-regulate activity detected at 92kDa, corresponding to pro-MMP-9, while no change to cleaved MMP-2 activity was appreciated (64 kDa band). Densitometric analyses of seven experiments were further corrected according to total protein extracted from tissue. C= control samples, α= IL-1α-treated samples, β= IL-1β-treated samples.
